# Efficient Determination of Water/Ice Phase Diagram through Isenthalpic–Isobaric Molecular Dynamics Simulations

**DOI:** 10.1021/acs.jpcb.5c01289

**Published:** 2025-04-20

**Authors:** Arthur Benigno Weidmann, Luís Fernando Mercier Franco, Amadeu K. Sum, Pedro de Alcântara Pessôa Filho

**Affiliations:** † Departamento de Engenharia Química, Escola Politécnica, Universidade de São Paulo (USP), Av. Prof. Luciano Gualberto, 380, São Paulo, São Paulo 05508-010, Brazil; ‡ Faculdade de Engenharia Química, Universidade Estadual de Campinas (UNICAMP), Av. Albert Einstein, 500, Campinas, São Paulo 13083-852, Brazil; § Chemical and Biological Engineering Department, Phases to Flow Laboratory, Colorado School of Mines, 1500 Illinois St., Golden, Colorado 80401, United States

## Abstract

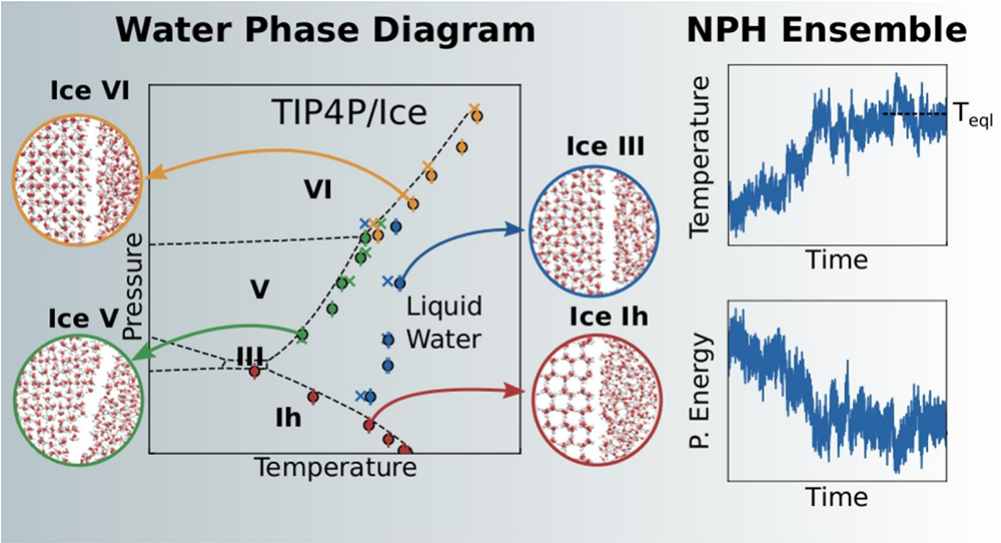

Predicting the water phase diagram is a powerful way to evaluate water models through molecular simulations. Equilibrium points are usually obtained through free energy calculations or direct coexistence simulations in the *NPT* ensemble. The former can be complex, especially for ice with partial proton order, while the latter can require multiple long and computationally costly simulations. In this work, we report the melting points of ice Ih, III, V, and VI between 0.1 and 1190 MPa through molecular dynamics direct coexistence simulations in the *NPH* ensemble. Our results are consistent with the original TIP4P/Ice work coexistence lines, except for ice III, for which we report a much larger stability region. Our data agree with recent works, validating this methodology as an alternative to free energy calculations and *NPT* direct coexistence simulations for high-pressure phases of ice.

## Introduction

Water is a molecule of deep interest for researchers, not only because of its general importance, but also for its complex behavior. The phase diagram of water presents a vapor phase, a liquid phase with numerous anomalies^1,2^ (including hypothesized phases of supercooled water^2^), and 20 recognized ice polymorphs.^3^ Its determination through molecular simulations is a useful way to evaluate the capability of water models to reproduce experimental behavior.^4,5^

The TIP4P^6^ model and its variants are some of the most commonly used water models. Among these variants, the TIP4P/2005,^7^ reparametrized as a general model of water, and the TIP4P/Ice,^8^ reparametrized to correctly reproduce the melting point of ice Ih, are specially well suited to generate the phase diagram. The first determination of the complete phase diagram of water was published by Sanz et al.^4^ in 2004, for the TIP4P and SPC/E^9^ models. Since then, other models were used to draw the phase diagram.^5,10−18^ Concerning the solid–liquid equilibrium, some works have focused on the determination of the melting point of ice Ih.^19−25^

The phase diagram of water is commonly built by first determining individual equilibrium points through either free-energy calculations or direct coexistence simulations.^14,18^ Thermodynamic integration can be performed through free energy calculations, but the simulation of solid phases require particular techniques, namely the Einstein crystal method^26^ or its variant, the Einstein molecule method.^27,28^ A major distinction among ice phases is the proton disorder. Ice Ih, VI, and VII present full proton disorder, while ice II, VIII, IX, XI, XIII, XIV, and XV have ordered protons. Ice III and V, on the other hand, present a higher complexity with their partial proton disorder.^14^ The free-energy calculation for proton-ordered ice is straightforward, whereas proton-disordered ice requires an added Pauling entropy contribution,^29^ and ice with partial proton disorder require a different treatment, usually an entropy contribution approximation.^30^ Macdowell et al.^30^ addressed this fact by adding a modified Pauling entropy.

The direct coexistence method (DCM), initially applied by Ladd and Woodcock,^31,32^ provides a simpler option based on the simulation of solid and fluid phases in coexistence. The procedure to map the phase diagram relies on the evolution of the system at each individual condition regarding its phases using a specific ensemble. The most commonly used isothermal ensembles such as *NVT* and *NPT* result in a single solid or fluid phase system. Several simulations at the same pressure are required to verify the equilibrium temperature between the conditions for which ice growth or melting dominates the simulation box, which can be computationally costly.

The less commonly used *NPH* ensemble is an alternative that can provide a phase diagram point from a single simulation. In the absence of a thermostat, the released enthalpy of formation or dissociation of the solid shifts the temperature of the system to the equilibrium temperature and fluctuates around it.^11^ The *NPH* ensemble has been used for melting temperature determination of some water models^20,22,23,33,34^ and other systems, such as Cu,Ni, and Al,^35^ silicon,^36,37^ Lennard-Jones fluids^38^ and bromine clathrate polymorphs.^39^ The interface between ice and clathrate hydrates^40^ and the thermal dissociation of gas hydrates^41^ have also been studied.

Once individual equilibrium points are determined, a Gibbs–Duhem integration^42^ can be performed to reproduce each coexistence line. As an advantage over the thermodynamic integration technique, the direct coexistence method (DCM) accounts for the proton disorder entropic contribution with actual dynamic simulations of the solid phase,^14,18^ and no significant effect of the initial proton order appears to be present.^14^ Conde et al.^14^ pointed out that the parameters that represent the degree of proton disorder might be different for experiments and models employed in free-energy calculations. The works of Conde et al.^14^ and Bore et al.^18^ support this claim by estimating the coexistence of ice III for the TIP4P and TIP4P/Ice models, respectively, ∼25 K above previous free energy results. A significant larger stability region for ice III is verified, along with a smaller ice V stability region.

In a previous study,^43^ we detailed a simpler methodology of using the DCM in the *NPH* ensemble to determine the dissociation temperature of gas hydrates. In this work, we applied the same methodology to determine the melting temperature of ice Ih, III, V, and VI for the TIP4P/Ice model, with the aim of obtaining correct values for ice with partial proton order through less complex simulations.

## Computational Methods

**Figure 1:**
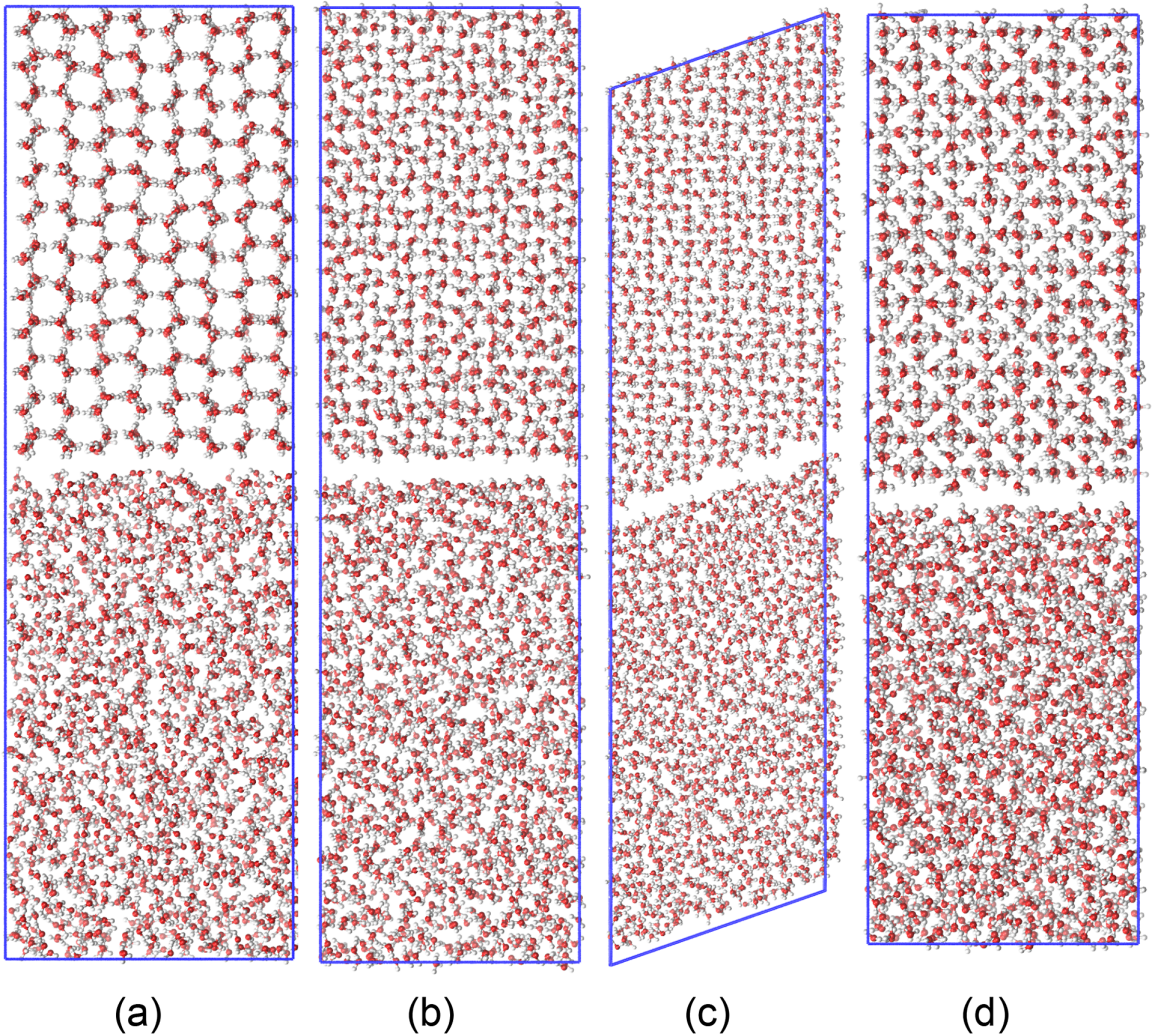
Initial configuration of (a) ice Ih, (b) ice III, (c) ice V, and (d) ice VI simulation boxes. Solid phases on the top and liquid water phases on the bottom. Water molecules are shown in white (hydrogen) and red (oxygen). Virtual sites of water are omitted.

The simulations were performed in GROMACS^44^ using the TIP4P/Ice^8^ model. The ice phases were generated using GenIce2.^45^ Each solid phase was built with ∼1500 water molecules. The simulation boxes built are shown in Figure 1.

Equilibration of each single-phase ice was performed anisotropically for each pressure condition to equilibrate the solid. The same number of water molecules was added to each box as a liquid phase. The exact number of molecules in the ice phase, number of unit cells, pressure ranges, and number of points simulated for each polymorph are presented in Table 1.

**Table 1:** Number of Water Molecules in Solid Phase, Number of Unit Cells, Pressure Range and Number of Points Simulated for Each Ice Phase

phase	number of molecules	unit cells	pressure range [MPa]	number of points
Ice Ih	1536	6 × 4 × 4	0.1–290	6
Ice III	1344	4 × 4 × 7	200–800	5
Ice V	1512	6 × 3 × 3	420–770	5
Ice VI	1500	5 × 5 × 6	780–1180	5

The two phase box was minimized using the steepest descent algorithm and equilibrated in an *NPT* ensemble for 200 ps, with the barostat applied only in the direction orthogonal to the interface. The temperature and pressure were controlled using the V-rescale thermostat and the Berendsen barostat, respectively. The production followed for 100 ns in the *NPH* ensemble with the Parrinello–Rahman barostat, applied only in the direction orthogonal to the interface. A time constant of 8 ps was used. No position restraints were used during the equilibration to avoid their effects and possible artifacts in the production trajectories.

The equations of motion were integrated using a leapfrog algorithm. The LINCS algorithm constrained the hydrogen atom bonds, and no long-range corrections were applied. Both the short-range van der Waals and long-range electrostatic interactions were computed using the smooth particle mesh Ewald (PME) summation algorithm. Particle-Mesh-Ewald calculations were used for the electrostatic contributions due to the lack of long-range corrections in a two-phase inhomogeneous system. The overall procedure to ensure energy conservation was similar to that previously used,^43^ even though preliminary simulations seemed to converge without the optimized parameters from that work. Thus, all production simulations were run with double precision, with a time step of 1 fs. The neighbor list was updated every 10 steps, and the Verlet buffer tolerance parameters used were given by GROMACS as an output of the *grompp* command. The final temperature was computed as the average of the last 25 ns of simulation. The error was determined through a block average calculation (described in the Supporting Information). Trajectories were visualized with VMD.^46^

## Results and Discussion

**Figure 2:**
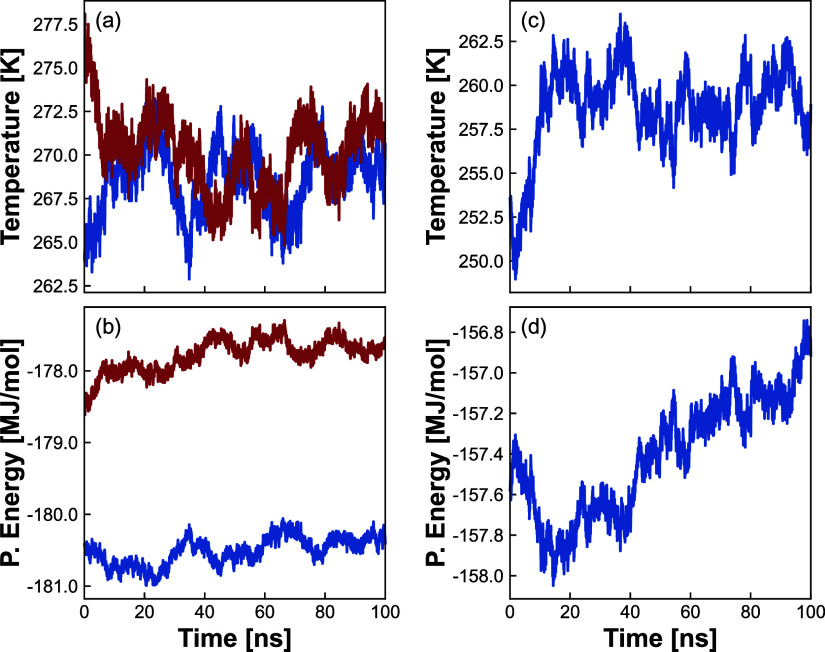
(a) Temperature and (b) potential energy evolution for ice Ih *NPH* ensemble simulations at 0.1 MPa. Simulations departing from an upper and a lower temperature in relation to the equilibrium are shown in red and blue, respectively. (c) Temperature and (d) potential energy evolution for ice III *NPH* ensemble simulations at 200 MPa. Curves are smoothed by a moving average window equivalent to 600 ps.

The size of each phase is an important aspect for keeping the phase coexistence as the ice phase grows or melts, since a simulation in the *NPH* ensemble is not useful if no phase equilibrium exists. The system size choice has also been shown to affect the obtained melting point error when the DCM method is applied. Conde et al.^24^ have determined errors of 1.5, 0.5, and 0.1 K for the direct coexistence melting temperature determination in the *NPT* ensemble for ice Ih systems of 1024, 2000, and 8000 water molecules, respectively. In this work, we simulated ice phases of every polymorph with ∼1500 water molecules, as a compromise between a smaller error and a lower computational cost. An example of the temperature and potential energy evolution in an *NPH* simulation is shown in Figure 2 for ice Ih at 0.1 MPa and for ice III at 200 MPa.

To demonstrate the convergence of the method, two different initial temperatures, one below and one above the expected equilibrium temperature, were simulated for ice Ih systems. The red curve plotted in Figure 2a demonstrates a decrease in temperature as the system starts at a temperature above the equilibrium (∼275 K), because of the enthalpy absorbed by the melting of the ice. Similarly, the blue curve shows the increase in temperature due to enthalpy release by ice growth, as it starts at ∼265 K, below the equilibrium temperature. Both curves converge and oscillate around the equilibrium temperature of ∼270 K. The potential energy curves in Figure 2b approximately mirror the temperature curves, with no significant energy drift. We achieve convergence of the two curves to the equilibrium temperature in less than ∼20 ns for ice Ih systems, as can be observed in the example shown in Figure 2a.

**Figure 3:**
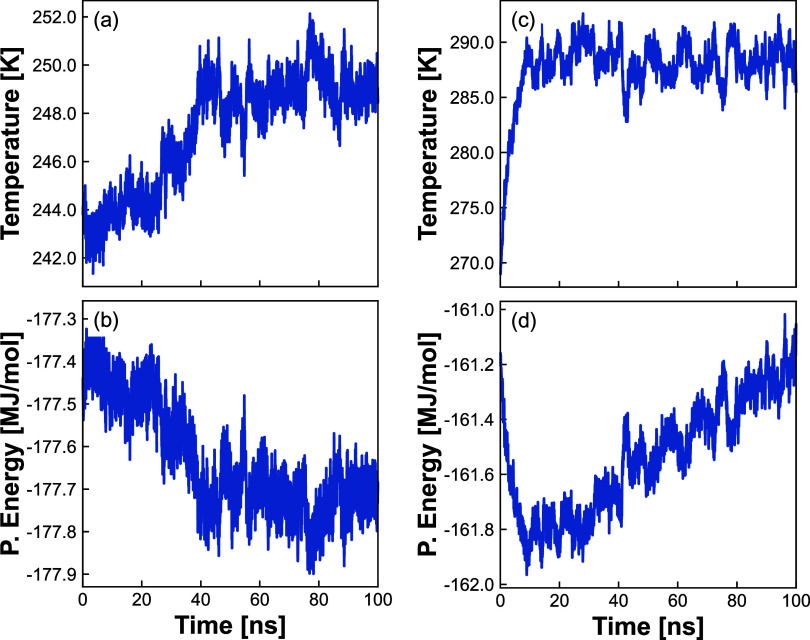
(a) Temperature and (b) potential energy evolution for ice V *NPH* ensemble simulations at 510 MPa. (c) Temperature and (d) potential energy evolution for ice VI *NPH* ensemble simulations at 1190 MPa. Curves are smoothed by a moving average window equivalent to 600 ps.

Single simulations were performed for ice III, V, and VI. Figure 2c shows for ice III at 200 MPa a clear behavior of increasing temperature (∼10 K) in the initial 20 ns of simulation, followed by an oscillatory behavior around the estimated equilibrium temperature of ∼260 K for the remaining 80 ns. Other ice phases with a single simulation required lengths up to 100 ns to verify the equilibrium temperature. An energy drift can be observed in Figure 2d through the rise in the potential energy with no mirrored decrease in temperature. We deem this drift as acceptable due to the normal behavior of the system and its temperature. It is also expected that some energy drift should happen, since the Verlet buffer tolerance temperature in GROMACS only aims to conserve energy within a limit. An example of the temperature and potential energy evolution is shown in Figure 3 for ice V at 510 MPa and for ice VI at 1190 MPa.

Figure 3a shows for ice V at 510 MPa a longer simulation time needed (∼40 ns) for the temperature to reach equilibrium and oscillate around it, even for a smaller temperature range of ∼7 K when compared to the previous example of ice III in Figure 2c. The potential energy curve in Figure 3b exhibits no energy drift. As shown in the Supporting Information, ice V at 420 MPa was an exception for the simulation time, as it was necessary to extend the simulation until 125 ns to verify the equilibrium temperature.

Figure 3c presents the behavior of ice VI at 1190 MPa with a complete rise in temperature in ∼10 ns. We highlight that the range in temperature covered is ∼20 K, showing the method’s capability to be applied to other systems with poor estimates of equilibrium temperature used as initial guesses. The potential energy evolution presented in Figure 3d is similar to Figure 2d and the same considerations can be made. The simplicity of an ice system when compared to a gas hydrate system (multicomponent and triphasic) is noted, since these systems might require ∼500 ns in the *NPH* ensemble and microsecond long simulations in the *NPT* or *NVT* ensembles to converge.^43^ No significant energy drift was observed in any of the simulations for ice.

Bore et al.^18^ proposed an enhanced sampling method to obtain the equilibrium points as opposed to running direct coexistence simulations that result in the irreversible melting or growth of the ice phase (*NPT* or *NVT* ensembles), resulting in a single-phase system. Although their method was applied with shorter simulations, our work shows that the same equilibrium points can be obtained in reasonably short molecular dynamics simulations in the *NPH* ensemble using a less complex methodology. Table 2 presents the dissociation temperature values obtained in this work for Ice Ih, III, V, and VI.

**Table 2:** Ice Polymorphs Dissociation Temperature Values Obtained in This Work through the *NPH* Ensemble

ice	pressure [MPa]	temperature [K]
Ih	0.1	270 ± 1
Ih	10	269 ± 1
Ih	50	264 ± 1
Ih	100	259 ± 1
Ih	200	244 ± 1
Ih	290	228 ± 1
III	200	260 ± 1
III	310	264 ± 1
III	400	264 ± 1
III	600	268 ± 1
III	800	267 ± 1
V	420	241 ± 1
V	510	249 ± 1
V	600	252 ± 1
V	690	257 ± 1
V	760	258 ± 1
VI	770	262 ± 1
VI	880	271 ± 1
VI	980	276 ± 1
VI	1080	284 ± 1
VI	1190	288 ± 1

The error presented in Table 2 was obtained through a block average calculation, described in the Supporting Information.

**Figure 4:**
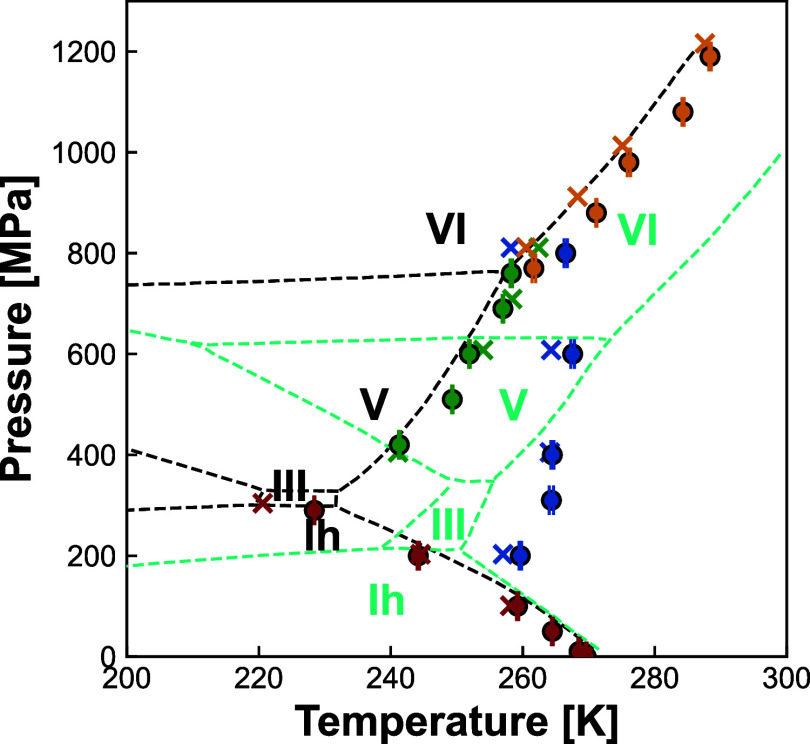
Phase coexistence data points from simulations. The results of this work are shown in circles, compared to Bore et al.^18^ as crosses. Data for ice Ih, III, V, and VI are represented by red, blue, green and yellow symbols, respectively. The phase diagram for the TIP4P/Ice model initially proposed by Abascal et al.^8^ is plotted in black, while the experimental phase diagram of water^47^ is plotted in cyan. The block average error calculated for our data (described in the Supporting Information) might appear smaller than the data point itself.

Figure 4 shows the comparison of the data points computed in this work (*NPH* simulations) with the experimental phase diagram of water, the phase diagram proposed by Abascal et al.^8^ for the TIP4P/Ice model (free energy calculations) and the points obtained by Bore et al.^18^ (enhanced sampling methodology).

Our data points for ice Ih, V, and VI (circles) are consistent with both the initially proposed phase diagram for TIP4P/Ice^8^ (black dashed lines) and those from Bore et al.,^18^ obtained through enhanced sampling simulations (crosses). The ice Ih data also agree with the experimental phase diagram (gray lines) at higher temperatures. The ice Ih melting points obtained by our methodology and by the methodology of Bore et al.^18^ go into the ice III stability region proposed for the TIP4P/Ice model at lower temperatures.^8^

Because of proton disorder, the computed liquid water–ice III coexistence points occupy a much larger pressure range and lie ∼30 K above the phase diagram,^8^ as demonstrated by Bore et al.^18^ for TIP4P/Ice and by Conde et al.^14^ for TIP4*P*/2005. The extra complexity in free energy calculations to estimate the degree of proton disorder for partially ordered ice phases highlights the advantages of performing dynamic simulations instead of the Einstein crystal method calculations to obtain the initial equilibrium points. Molecular dynamics simulations can also be used to properly validate these calculations before performing Gibbs–Duhem integration to obtain the coexistence line.

## Conclusions

Molecular dynamics simulations in the *NPH* ensemble were performed for ice Ih, III, V, and VI to determine their melting points. A number of 5 or 6 pressures are simulated for each ice polymorph and the equilibrium temperature is averaged from the last 20 ns of simulation. Our results agree with the original TIP4P/Ice work,^8^ except for ice III, which presents a much larger stability range due to the partial proton order considerations taken for the free energy calculations in their work, which is also in agreement with Bore et al.^18^

In conclusion, we can determine correct coexistence data of liquid water and ice Ih, III, V, and VI for the TIP4P/Ice model through molecular dynamics simulations in the *NPH* ensemble. Although moderate system sizes and simulation lengths are necessary for the robustness of this methodology, the results show how this type of simulation can be a reliable alternative to free energy calculations and direct coexistence simulations in the *NPT* ensemble to determine the melting points of high-pressure ice polymorphs.

## Supporting Information Available

GROMACS mdp file for production simulations, temperature and potential energy evolution figures for every pressure condition, and error calculation procedure (PDF)
