# Supplementary material for: Efficient Determination of Water/Ice Phase Diagram through Isenthalpic–Isobaric Molecular Dynamics Simulations
Source: J Phys Chem B. 2025 Apr 20;129(19):4871–7. doi: 10.1021/acs.jpcb.5c01289 (PMC12086842; doi:10.1021/acs.jpcb.5c01289)
Supplement: Supplementary file 1 — jp5c01289_si_001.pdf [file jp5c01289_si_001.pdf]

SUPPORTING INFORMATION

Efficient Determination of Water/Ice Phase Diagram through Isenthalpic-Isobaric Molecular Dynamics Simulations

Arthur Benigno Weidmann,[†] Luís Fernando Mercier Franco,[‡] Amadeu K. Sum,[¶] and Pedro de Alcântara Pessôa Filho[*,†]

†*Universidade de São Paulo (USP), Departamento de Engenharia Química, Escola Politécnica, Av. Prof. Luciano Gualberto, 380, 05508-010, São Paulo, SP, Brazil*

‡*Universidade Estadual de Campinas (UNICAMP), Faculdade de Engenharia Química, Av. Albert Einstein, 500, 13083-852, Campinas, SP, Brazil*

¶*Phases to Flow Laboratory, Colorado School of Mines, Chemical and Biological Engineering Department, 1500 Illinois St., Golden, CO 80401*

E-mail: pedropessoa@usp.br

NPH simulations parameters

The *mdp* file GROMACS parameters used for the NPH production simulations are shown in Table S1.

Table S1: An example of the GROMACS *mdp* file parameters for production NPH runs. This in an example of a simulation at 0.1 MPa. The Verlet-buffer-tolerance parameter is provided for every configuration and condition by the *grompp* command.

Parameter	Value
integrator	md
dt	0.001
nsteps	100000000
nstxout	50000
nstvout	50000
nstenergy	200
nstlog	2000
nstxout-compressed	10000
nstlist	10
continuation	yes
constraint-algorithm	lincs
constraints	h-bonds
lincs-iter	2
verlet-buffer-tolerance	4.4e-07
cutoff-scheme	Verlet
coulombtype	PME
rcoulomb	1.0
fourierspacing	0.20
pme-order	6
vdwtype	PME
rvdw	1.0
DispCorr	no
pcoupl	Parrinello-Rahman
pcoupltype	anisotropic
tau_p	8.0
compressibility	4.5e-5 0.0 0.0 0.0 0.0 0.0
ref_p	1.0 0.0 0.0 0.0 0.0 0.0

Temperature and potential energy evolution curves

The temperature and potential energy evolution of every NPH simulation performed in this work is shown in Figures S1 to S20. Simulations departing from an upper and a lower temperature in relation to the equilibrium are shown in red and blue, respectively, for Ice Ih systems. Ice III, V, and VI individual curves are shown in blue. Curves are smoothed by a moving average window equivalent to 600 ps.

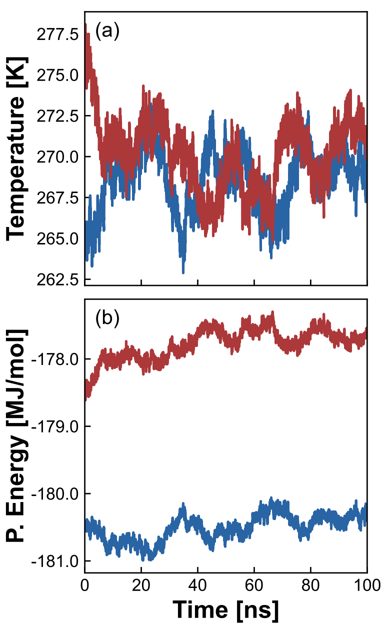

Figure S1: (a) Temperature and (b) potential energy evolution for Ice Ih NPH ensemble simulations at 0.1 MPa.

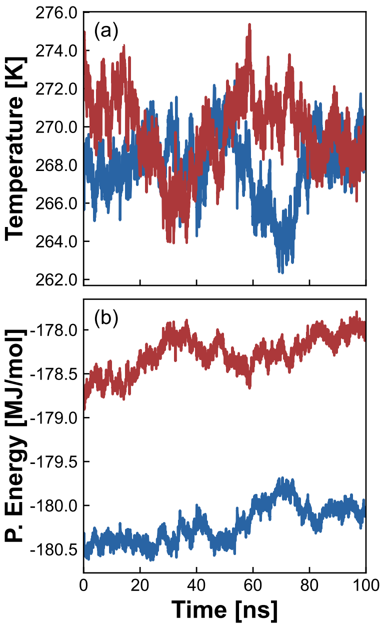

Figure S2: (a) Temperature and (b) potential energy evolution for Ice Ih NPH ensemble simulations at 10 MPa.

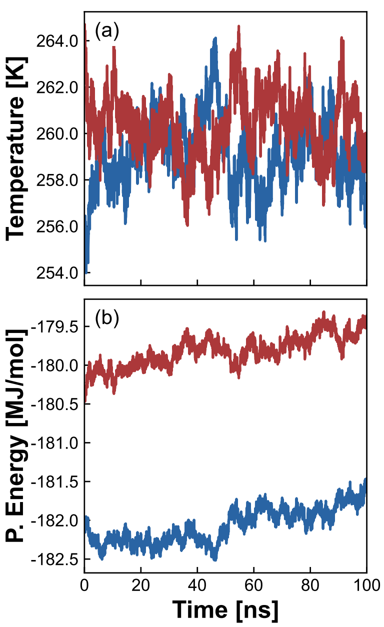

Figure S3: (a) Temperature and (b) potential energy evolution for Ice Ih NPH ensemble simulations at 100 MPa.

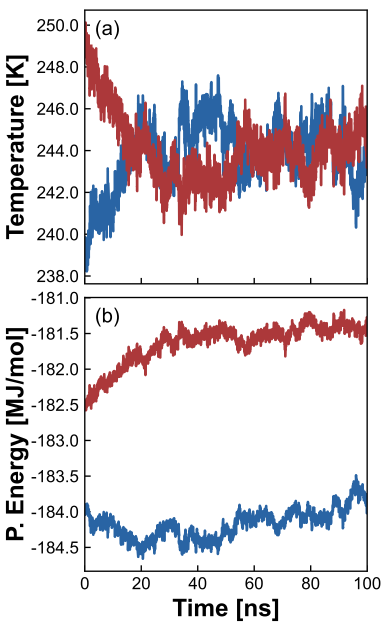

Figure S4: (a) Temperature and (b) potential energy evolution for Ice Ih *NPH* ensemble simulations at 200 MPa.

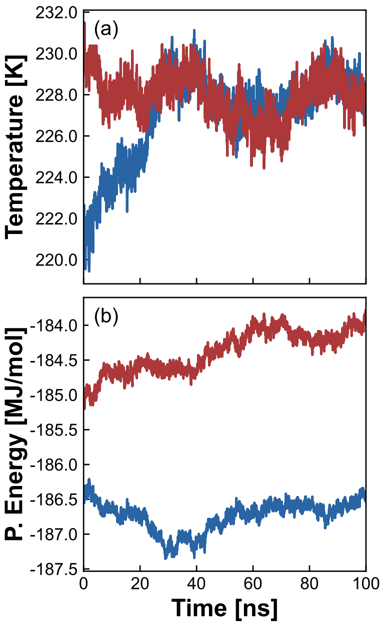

Figure S5: (a) Temperature and (b) potential energy evolution for Ice Ih *NPH* ensemble simulations at 290 MPa.

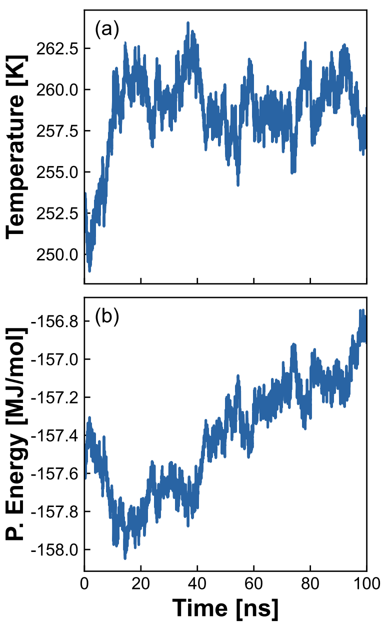

Figure S6: (a) Temperature and (b) potential energy evolution for Ice III *NPH* ensemble simulations at 200 MPa.

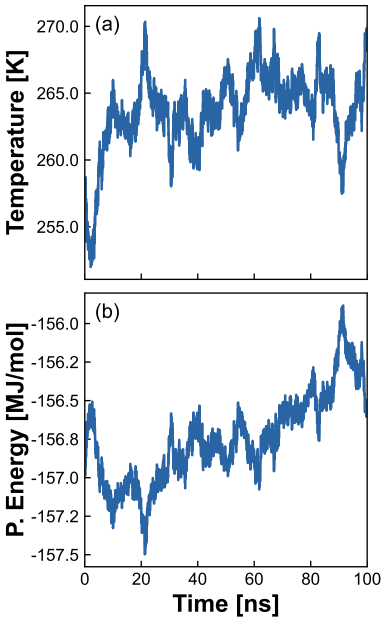

Figure S7: (a) Temperature and (b) potential energy evolution for Ice III *NPH* ensemble simulations at 310 MPa.

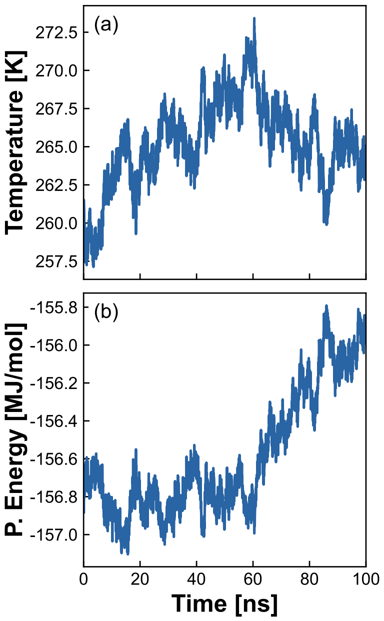

Figure S8: (a) Temperature and (b) potential energy evolution for Ice III *NPH* ensemble simulations at 400 MPa.

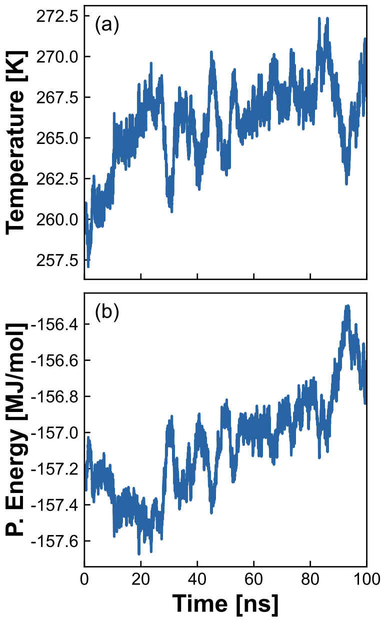

Figure S9: (a) Temperature and (b) potential energy evolution for Ice III *NPH* ensemble simulations at 600 MPa.

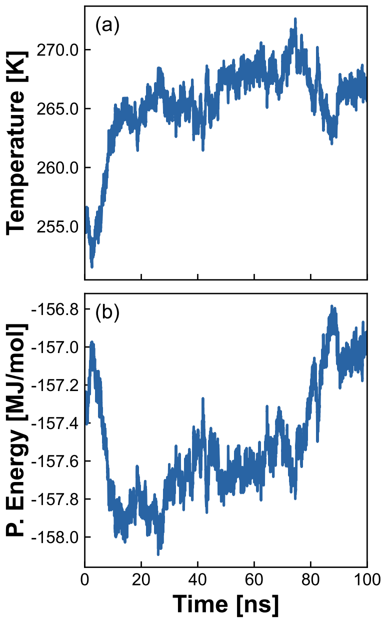

Figure S10: (a) Temperature and (b) potential energy evolution for Ice III *NPH* ensemble simulations at 800 MPa.

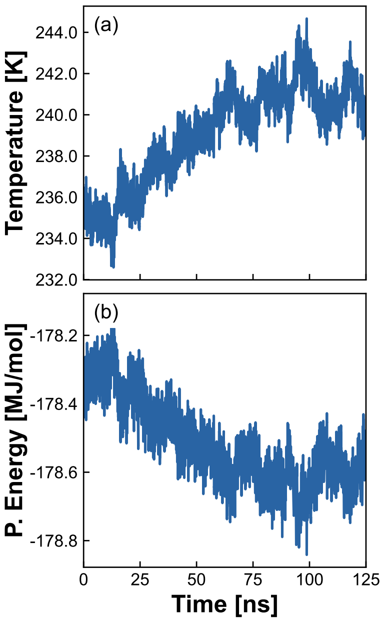

Figure S11: (a) Temperature and (b) potential energy evolution for Ice V *NPH* ensemble simulations at 420 MPa.

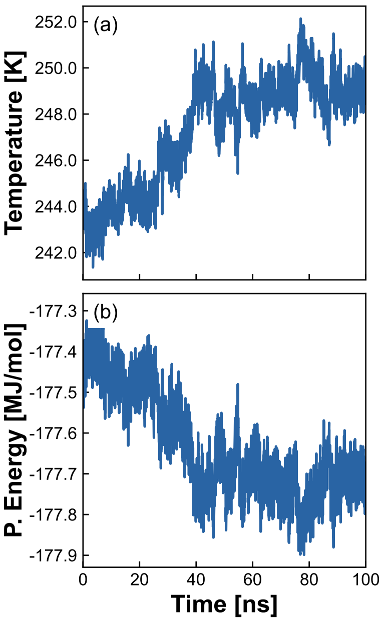

Figure S12: (a) Temperature and (b) potential energy evolution for Ice V *NPH* ensemble simulations at 510 MPa.

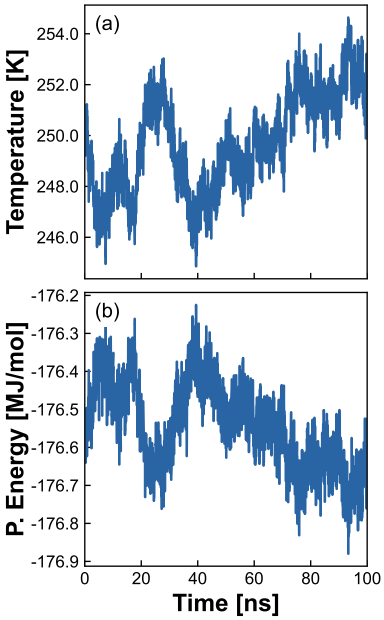

Figure S13: (a) Temperature and (b) potential energy evolution for Ice V *NPH* ensemble simulations at 600 MPa.

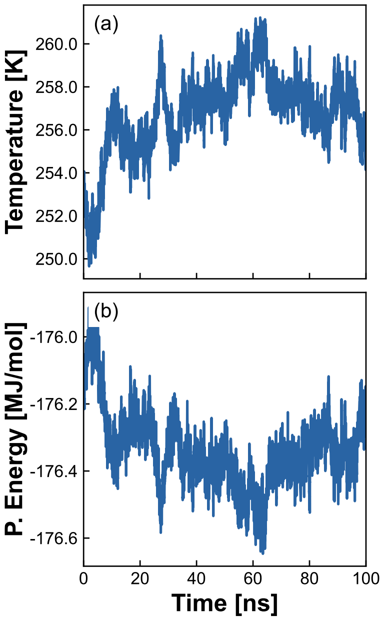

Figure S14: (a) Temperature and (b) potential energy evolution for Ice V *NPH* ensemble simulations at 690 MPa.

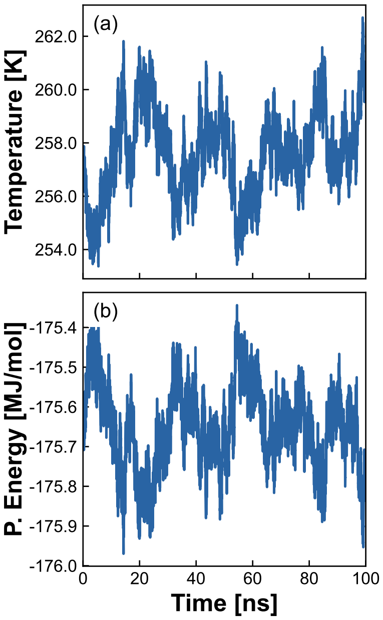

Figure S15: (a) Temperature and (b) potential energy evolution for Ice V *NPH* ensemble simulations at 760 MPa.

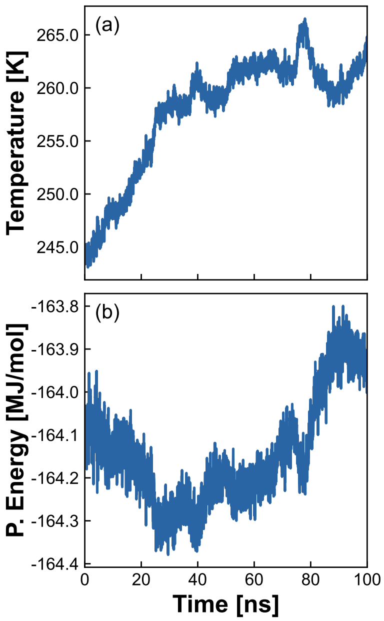

Figure S16: (a) Temperature and (b) potential energy evolution for Ice VI *NPH* ensemble simulations at 770 MPa.

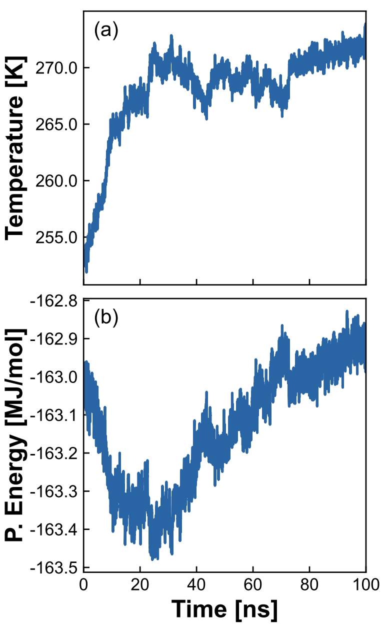

Figure S17: (a) Temperature and (b) potential energy evolution for Ice VI *NPH* ensemble simulations at 880 MPa.

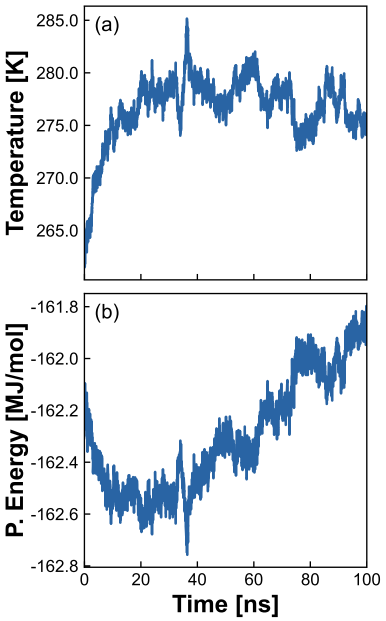

Figure S18: (a) Temperature and (b) potential energy evolution for Ice VI *NPH* ensemble simulations at 980 MPa.

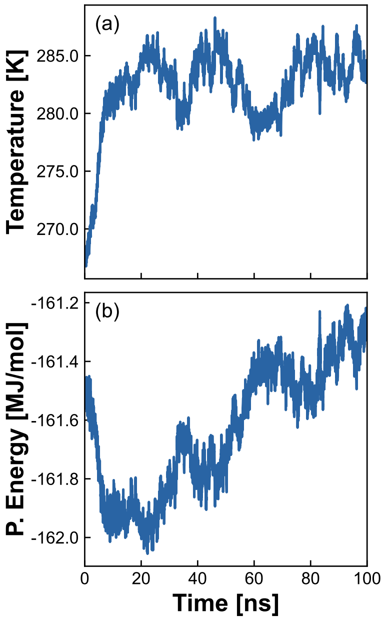

Figure S19: (a) Temperature and (b) potential energy evolution for Ice VI *NPH* ensemble simulations at 1080 MPa.

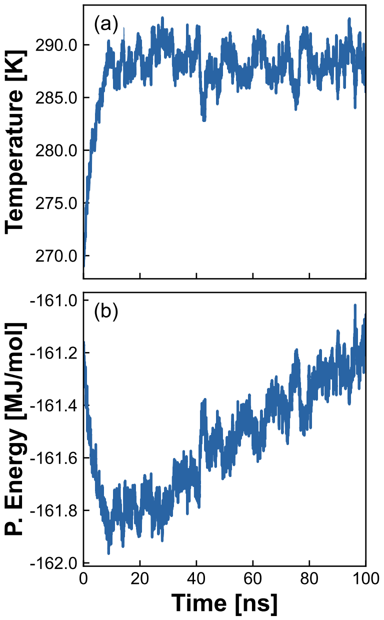

Figure S20: (a) Temperature and (b) potential energy evolution for Ice VI *NPH* ensemble simulations at 1190 MPa.

Block average error calculation for the temperature

The block average error calculation for the temperature values obtained are based on the equations shown in Allen and Tildesley.[1] The uncertainty is given by Equation (S1):

$$\sigma^2(\langle T \rangle_\tau) = s\frac{\sigma^2(T)}{\tau}, \tag{S1}$$

where τ is the total number of points (steps). $\sigma^2(T)$ is the variance and s is the statistical inefficiency, which are defined by Equations (S2) and (S3), respectively:

$$\sigma^2(T) = \frac{1}{\tau - 1}\sum_{t=1}^{\tau}(T_t - \langle T \rangle_t)^2, \tag{S2}$$

$$s = \lim_{\tau_b \to \infty} s(\tau_b), \tag{S3}$$

where $\tau_b = \tau / n_{\text{blocks}}$ and $s(\tau_b)$ is defined by Equation (S4):

$$s(\tau_b) = \frac{\tau_b \sigma^2(\langle T \rangle_b)}{\sigma^2(T)}, \tag{S4}$$

where $\sigma^2(\langle T \rangle_b)$ is the variance for the block average, given by Equation (S5):

$$\sigma^2(\langle T \rangle_b) = \frac{1}{n_{\text{blocks}} - 1}\sum_{b=1}^{n_{\text{blocks}}}(T_b - \langle T \rangle_t)^2. \tag{S5}$$

References

(1) Allen, M. P.; Tildesley, D. J. *Computer Simulation of Liquids*, 2nd ed.; Oxford University Press, 2017.
